# Validity of the older people quality of life-7 domains (OQoL-7) scale

**DOI:** 10.1186/s12955-020-01589-5

**Published:** 2020-10-14

**Authors:** Yves Henchoz, Christophe Büla, Idris Guessous, René Goy, Marc Dupuis, Brigitte Santos-Eggimann

**Affiliations:** 1 Centre for Primary Care and Public Health (Unisanté), University of Lausanne, Route de la Corniche 10, 1010 Lausanne, Switzerland; 2 Service of Geriatric Medicine and Geriatric Rehabilitation, University of Lausanne Hospital Centre, Lausanne, Switzerland; 3 Division of Primary Care Medicine, Department of Primary Care Medicine, Geneva University Hospitals, Geneva, Switzerland; 4 Pro Senectute Vaud, Lausanne, Switzerland; 5 Department of Medicine, Institute of Global Health, University of Geneva, Geneva, Switzerland

**Keywords:** Quality of life, Older people, Scale, Validity, Importance weighting

## Abstract

**Background:**

The Older people Quality of Life-7 domains (OQoL-7) is a 28-item multidimensional questionnaire developed to measure community-dwelling older people’s QoL. The OQoL-7 assesses both importance of and satisfaction in seven QoL domains (Material resources; Close entourage; Social and cultural life; Esteem and recognition; Health and mobility; Feeling of safety; and Autonomy). This study aimed to investigate concurrent and construct validity of the OQoL-7. A secondary aim was to compare different methods of weighting participants’ ratings of satisfaction according to their individual ratings of importance, as compared to the OQoL-7 total score (unweighted).

**Methods:**

Data came from the first and second samples of the Lausanne cohort 65+ study, assessed at the same age of 72–77 years in 2011 (N = 1117) and 2016 (N = 1091), respectively. To assess concurrent validity, the OQoL-7 was compared to other measures of the same concept (single QoL item) or related concepts (self-rated health, SF-12). Construct validity was tested by comparing subscores in the seven QoL domains in the presence and absence of two stressful events during the preceding year (financial difficulties and relationship difficulties). The effect of importance weighting was assessed using moderated regression analysis.

**Results:**

The OQoL-7 total score was significantly associated with the single QoL item (Spearman’s rho 0.46), self-rated health (Spearman’s rho 0.34), SF-12 physical (Spearman’s rho 0.22) and mental (Spearman’s rho 0.28) component scores. Large differences (Cohen’s d > 0.8) were observed in the presence or absence of stressful events in the expected QoL domains: “Material resources” in the presence or absence of “Financial difficulties” (Cohen’s d 1.34), and “Close entourage” in the presence or absence of “Relationship difficulties” (Cohen’s d 0.84). Importance weighting resulted in a very small improvement in the prediction of the single QoL item (ΔR^2^ 0.018). All results were highly consistent across 2011 and 2016 samples.

**Conclusions:**

The OQoL-7 showed adequate concurrent and construct validity in two samples of older people. In future studies, the decision to use weighted or unweighted scores will depend on the priority given to either optimizing the prediction of QoL or limiting the burden on respondents and the amount of missing data.

## Background

Since the beginning of quality of life (QoL) measurement half a century ago [1], plenty of instruments have been developed in various clinical or community settings, with a focus on different life stages. In older age, most research uses generic health-related QoL questionnaires, among which the most popular are the SF-36 [2] and the EQ-5D [3]. Facing the complexity and the multidimensional nature of QoL, more holistic approaches have led to the development of tools that consider multiple domains of older people’s QoL: the SEIQoL-DW [4], the LEIPAD [5], the CASP-19 [6], the WHOQOL-BREF [7] with its WHOQOL-OLD module [8], the EQOLI [9], and the OPQOL [10]. A single global rating of QoL can also be a valuable tool, particularly when the focus is on measuring QoL—broadly defined—rather than addressing each of its domains [11].

While several questionnaires are already available to measure older people’s QoL, there are some reasons for introducing a new one. First, modern societies are changing rapidly and it is questionable whether QoL domains that were important two or three decades ago are still relevant nowadays. Second, quantitative and qualitative research that has been conducted after the development of the aforementioned QoL instruments [12] enriched significantly our knowledge, with the potential to improve QoL assessment. Third, the format of available instruments may not be suitable for all purposes. For instance, some may be too long for use in population-based studies, whereas others provide a too limited amount of details on the respondents’ QoL profile. In the absence of a gold standard, a higher number of valid instruments is likely to offer a larger choice, and thus to increase the probability of meeting users’ needs.

The Older people Quality of Life-7 domains (OQoL-7) scale was developed on the basis of available evidence [12, 13] and the expertise of scientists, clinicians, and field experts. It measures both the perceived importance and the perceived discomfort or dissatisfaction regarding 28 aspects of the respondent’s QoL. An exploratory principal components analysis of answers to the importance items identified 7 QoL domains (Material resources; Close entourage; Social and cultural life; Esteem and recognition; Health and mobility; Feeling of safety; and Autonomy). This factorial structure was subsequently confirmed in a validation sample, with moderate correlations and adequate internal consistency within each domain [14]. Content validity was further supported by answers from over 5000 community-dwelling older adults to an open-ended question on factors important to their QoL that were potentially missing on the 28-item list [14]. A total of 303 (5.7%) respondents provided very sparse propositions, suggesting that no aspect of QoL that matters to most older persons was lacking.

The validity of the OQoL-7 scale has yet to be examined from several perspectives. First, it is necessary to ensure that it correlates with QoL measures obtained using other tools (concurrent validity). Second, a valid scale is expected to correlate with related constructs but not with dissimilar measures (construct validity). Third, since the OQoL-7 assesses both importance of and satisfaction in QoL domains, the relevance of both ratings remains to be clarified. As a step toward person-centered medicine, it seems intuitively desirable for a QoL questionnaire to take into account the extent to which its items are deemed important by individuals. The literature reveals various attempts to weigh satisfaction by importance. In a narrative review, Russel and Hubley concluded that weighted scores failed to receive significant empirical support [15]. Yet, completely abandoning importance weighting may be premature, as existing studies were criticized precisely for their insufficient variety of weighting methods and limited statistical power [16].

The present study aimed to investigate the concurrent and construct validity of the OQoL-7. A secondary aim was to analyze different methods of weighting participants’ ratings of satisfaction by ratings of importance, as compared to unweighted QoL.

## Methods

### Study design and population

The Lausanne cohort 65+ study (Lc65+) is a population-based study initiated in 2004 to investigate the frailty process in old age [17]. It involves three representative samples of the community-dwelling population in Lausanne (the capital of Canton Vaud, Switzerland) born before (1934–1938, N = 1564), during (1939–1943, N = 1489), and at the end of the Second World War (1944–1948, N = 1678). The present study focused on the first and second Lc65+ samples, enrolled at age 65–70 years in 2004 and 2009, respectively. We used data from a postal questionnaire and an in-person interview completed in 2011 (sample enrolled in 2004) and 2016 (sample enrolled in 2009). The postal questionnaire included an in-depth assessment of quality of life, thereby allowing to determine results’ consistency across two samples assessed at the same age range (72–77 years). Persons living in institutions or who did not answer in person (i.e. proxy respondent) were excluded.

### Measures

All measures were collected by postal questionnaire, except the Medical Outcomes Study 12-item Short Form Health Survey (SF-12) as well as income, which were collected during an in-person interview.

#### Older people Quality of Life-7 domains (OQoL-7)

The OQoL-7 is a 28-item questionnaire that was developed to assess the multidimensional QoL of community-dwelling older people. The questionnaire begins with a brief description of QoL to ensure that respondents have a common understanding of the construct (see Additional File 1). Respondents are then asked to rate each item on its perceived importance for their own QoL (very low; quite low; quite high; very high) as well as on potential discomfort or dissatisfaction currently perceived (not at all; a little; a lot). Answers to perceived discomfort or dissatisfaction on the 28 QoL items are reverse coded to express satisfaction (not at all = 2, a little = 1, a lot = 0) and then summed up. This total is divided by the maximum possible total (number of completed items multiplied by 2) and then multiplied by 100 to yield a QoL score ranging from 0 to 100, with higher scores indicating higher QoL. The same procedure is applied on the constituent items of each QoL domain to obtain its specific subscore. The QoL score is considered as missing if more than half of the items are missing. For each of the seven domains, the QoL subscore is considered as missing if more than one of its constituent items are missing. A previous article focused on importance ratings [14], which were used in the present study only for weighting purposes (see paragraph “Weighting procedures” section).

#### Single QoL item and self-rated health

Overall QoL was assessed by a single-item measure: “How do you rate your current quality of life? (excellent, very good, good, fair, poor)”. A single global rating of QoL is a simple and suitable instrument to measure QoL in a broad sense [11]. Self-rated health was reported as very good, good, average, poor, or very poor. A single question is a valuable indicator of the overall health status, and a strong predictor of morbidity and mortality [18, 19].

#### SF-12

The SF-12 v2 was completed during an in-person interview conducted by trained medical research assistants. Norm-based physical (PCS) and mental (MCS) component scores were obtained using linear transformations (mean = 50; SD = 10) [20]. Norm-based scores of the eight SF-12 dimensions were also calculated (General Health, Physical Functioning, Role Physical, Role Emotional, Bodily Pain, Vitality, Mental Health, and Social Functioning).

#### Stressful events (last 12 months)

Twenty events from the geriatric adverse events life scale (GALES [21]) were selected for their suitability in old age. Respondents were asked whether they faced any of these stressful events during the previous 12 months.

#### Socio-demographic characteristics

Information about respondents’ age and sex were obtained from the Population Office at the stage of study sampling and recruitment. Additional information was gathered by means of a postal questionnaire that provided information about highest level of education achieved (Basic compulsory (International Standard Classification of Education (ISCED [22]) level 0–2); Apprenticeship (ISCED level 3); Baccalaureate/professional degree (ISCED level 4–5); University/high school (ISCED level 6–8)) and living arrangement (Alone; With spouse (married or not); Other living arrangement). Information on household gross monthly income was collected during the in-person interview conducted by trained medical research assistants. It was divided by the household size (collected by postal questionnaire) to calculate gross monthly income.

### Analyses

#### Data quality

The proportion of missing values was calculated for each QoL item, for the seven domain subscores and for the QoL score. The proportion of participants at the minimum (floor) and maximum (ceiling) values was also calculated.

#### Concurrent validity

In the absence of a gold standard, the OQoL-7 was compared to other measures of the same concept (single QoL item) or related concepts (self-rated health, SF-12). Spearman correlations were calculated between the QoL score and the seven QoL subscores on the one hand, and the single QoL item, self-rated health, SF-12 PCS, and SF-12 MCS, on the other hand. These analyses were conducted on the 2011 sample and repeated on the 2016 sample. Correlations were interpreted as small (> 0.1), medium (> 0.3), or large (> 0.5) [23].

#### Construct validity

The OQoL-7 was tested for construct validity against stressful events during the previous 12 months. Whereas most of these events are likely to influence several QoL domains simultaneously, two events were analyzed more in depth based on their expected impact on a specific QoL domain. A strong association was expected between (1) “Financial difficulties” and the QoL domain “Material resources”; and (2) between Separation, Divorce, or Other difficulties in the couple (labelled “Relationship difficulties”) and the QoL domain “Close entourage”. Spearman correlations were calculated between stressful events and QoL subscores in the seven domains. In addition, the mean and standard deviation of QoL subscores in the seven domains were calculated in the presence and absence of both stressful events. The effect size was estimated using Cohen’s d and was interpreted as small (> 0.2), medium (> 0.5), or large (> 0.8) as proposed by Cohen [23]. To estimate 95% confidence intervals (CI) for Cohen’s d, bootstrapping was performed using 200 bootstrap replications.

To further assess construct validity, Spearman’s correlations were calculated between each of the 28 satisfaction QoL items and QoL subscores in the seven domains. In this particular analysis, for each item, its corresponding QoL subscore was recalculated without the item itself (i.e. considering only the other items in the domain concerned). It was expected that each item would be most strongly associated with its corresponding domain.

#### Weighting procedures

In all analyses assessing data quality, concurrent validity, and construct validity, the OQoL-7 total score and the seven subscores were calculated without taking into account importance ratings. In the following analyses, four different weighting procedures were performed, each consisting in the multiplication of importance and satisfaction scores, divided by the sum of importance scores. In the first three methods, satisfaction was weighted by importance at the item level and importance was coded in three different ways. First, items whose importance was rated very low were given a zero weight (weighted QoL score 1: very low = 0; quite low = 1; quite high = 2; very high = 3). The particularity of this method is that items deemed of very low importance have no influence on the total score. Second, items importance had still a linear increasing weight (weighted QoL score 2: very low = 1; quite low = 2; quite high = 3; very high = 4). This method makes the assumption of constant intervals between importance ratings. Third, items importance had a quadratic increasing weight (weighted QoL score 3: very low = 1; quite low = 4; quite high = 9; very high = 16). Like in method 2, the influence of items on the total score increases as their importance increases, but this increase is not linear. In other words, in method 3 the total score is even more influenced by items with a high importance than in method 2. In the fourth method (weighted QoL score 4), satisfaction was weighted by importance at the domain level. The seven domains’ satisfaction subscores were first multiplied by their respective importance subscores according to participant’s rating, then summed-up, and finally divided by the sum of importance subscores. Spearman correlations were calculated between weighted and unweighted QoL scores, and between weighted QoL scores and the single QoL item. In addition, moderated regression analysis was conducted [16]. It consisted in regressing the single QoL item on satisfaction subscores of the seven QoL domains (step 1), then additionally on importance subscores of the seven QoL domains (step 2), and on the seven interaction terms of satisfaction by importance (step 3). A significant increase in explained variance (R^2^) from one step to the next step was tested using an F-test. The magnitude of this increase was interpreted as small (> 0.02), medium (> 0.13), or large (> 0.26) as proposed [23].

## Results

**Fig. 1:**
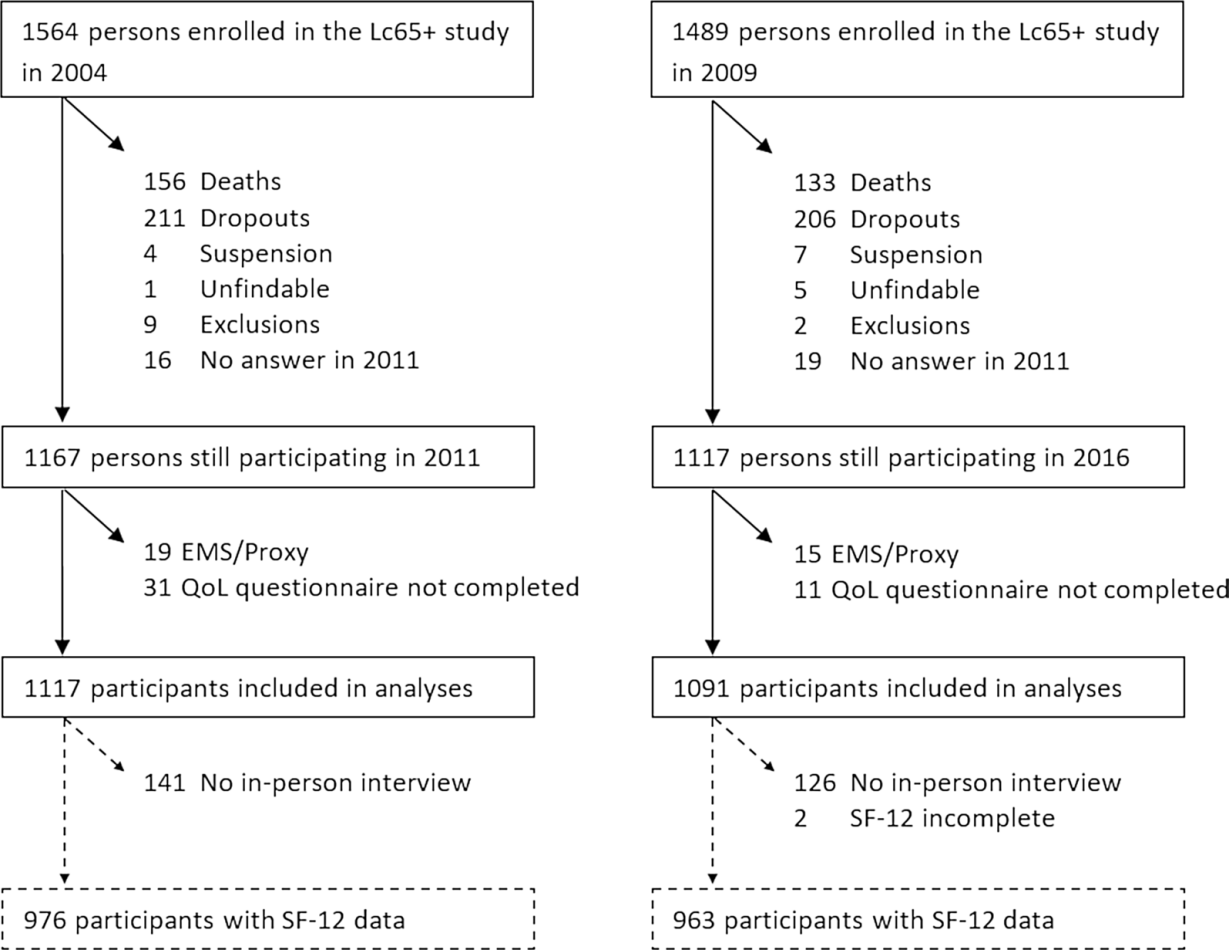
Flow diagram of participants' selection process

Figure 1 shows the flow diagram of participants included in the present study. Analyses were performed on a total of 1117 participants in 2011 and 1091 participants in 2016. Analyses including SF-12 data were performed on a total of 976 participants in 2011 and 963 participants in 2016. This difference is due to participants who completed the postal questionnaire but did not attend the in-person interview. Table 1 displays the main characteristics of the two samples. Of these, the majority were women, age ranged from 73 to 77 years, four in ten participants reported apprenticeship as the highest level of education achieved, and most of them were living with others. The QoL and health characteristics of both samples can be found in Additional File 1: Table S1.

**Table 1:** Characteristics of the 2011 and 2016 samples

Characteristics	Sample 2011 (N = 1117)	Sample 2016 (N = 1091)
Age, years		
Range	73–77	73–77
Mean (SD)	75.0 (1.4)	74.8 (1.4)
Sex, N (%)		
Women	674 (60.3%)	672 (61.6%)
Men	443 (39.7%)	419 (38.4%)
Education,^a^ N (%)		
Basic compulsory	258 (23.2%)	185 (17.0%)
Apprenticeship	451 (40.6%)	436 (40.0%)
Baccalaureate/Prof. degree	266 (23.9%)	278 (25.5%)
University/high school	136 (12.2%)	192 (17.6%)
Gross monthly income, N (%)		
< 2500 CHF	205 (23.3%)	227 (24.6%)
2500 to < 5000 CHF	500 (56.8%)	514 (55.8%)
5000 to < 7500 CHF	142 (16.1%)	140 (15.2%)
> 7500 CHF	34 (3.9%)	41 (4.5%)
Living arrangement, N (%)		
Alone	442 (39.8%)	496 (45.6%)
With spouse (married or not)	609 (54.9%)	534 (49.0%)
Other living arrangement	59 (5.3%)	59 (5.4%)

CHF Swiss francs

^a^See text

### Data quality

The QoL score was missing in less than 2% of participants from both 2011 (N = 1117) and 2016 (N = 1091) samples (see Additional File 1: Table S2). Only one participant from the 2011 sample reached the minimal QoL score, while around one in six participants reached the maximal QoL score (i.e. ceiling effect) in both 2011 and 2016 samples. At the domain level, less than 3% of participants reached the minimum subscore in any domain, whereas about half to two thirds reached the maximum subscores (45.2–61.2% in 2011; 48.7–66.3% in 2016). At the item level (see Additional File 1: Table S3), the proportion of participants reporting the least favorable answer choice was below 8% for all items, whereas the proportion of participants reporting the most favorable answer choice ranged from 53.7 to 87.7% in 2011, and from 60.1 to 88.7% in 2016. The proportion of missing items was below 7%, except the item “Couples’ relationships” that was missing for one in four participants. The SF-12 PCS and MCS had no floor or ceiling effects. However, among the eight dimensions, one showed a notable floor effect (vitality, 14.0%) and five showed a notable ceiling effect (Physical Functioning 54.8%; Role Physical 55.0%; Role Emotional 56.6%; Bodily Pain 51.3%; Social Functioning 65.3%).

### Concurrent validity

As expected, the QoL score was most strongly associated with the single QoL item, and was also significantly associated with the other constructs (i.e. self-rated health, physical health and mental health, all *P* < 0.001, see Table 2). A close to large correlation was observed with the single QoL item, whereas the correlations were small to medium with the other constructs. The seven QoL subscores were also significantly associated with the single QoL item (medium correlations). Among the seven domains, “Health and mobility” had the strongest correlations with self-rated health (close to large correlation) and with SF-12 PCS (medium correlation), as expected. Five domains (Material resources, Close entourage, Social and cultural life, Esteem and recognition, Autonomy) correlated higher with SF-12 MCS than with SF-12 PCS. These results were highly consistent across 2011 and 2016 samples.

**Table 2:** Concurrent validity (Spearman’s rho)

	Single QoL item		Self-rated health		SF-12 (PCS)		SF-12 (MCS)	
	2011 (N = 893)	2016 (N = 914)	2011 (N = 893)	2016 (N = 914)	2011 (N = 893)	2016 (N = 914)	2011 (N = 893)	2016 (N = 914)
QoL score	0.46***	0.47***	0.34***	0.34***	0.22***	0.26***	0.28***	0.33***
QoL subscores								
Material resources	0.33***	0.40***	0.19***	0.18***	0.08*	0.12***	0.12***	0.19***
Close entourage	0.30***	0.30***	0.17***	0.16***	0.06	0.10**	0.24***	0.22***
Social and cultural life	0.27***	0.30***	0.17***	0.23***	0.11***	0.20***	0.17***	0.25***
Esteem and recognition	0.30***	0.31***	0.22***	0.21***	0.11***	0.09***	0.23***	0.28***
Health and mobility	0.42***	0.41***	0.43***	0.42***	0.33***	0.35***	0.23***	0.25***
Feeling of safety	0.33***	0.29***	0.25***	0.24***	0.18***	0.19***	0.14***	0.21***
Autonomy	0.33***	0.27***	0.24***	0.21***	0.20***	0.15***	0.25***	0.20***

QoL quality of life

*P < 0.05; **P < 0.01; ***P < 0.001

### Construct validity

In the 2011 sample, significant differences in QoL subscores were observed in the presence or absence of stressful events during the previous 12 months in the expected specific domains (see Table 3). A large difference was found in the QoL domain “Material resources” in the presence or absence of “Financial difficulties” (Cohen’s d 1.34; 95% CI 1.02–1.66), and in the QoL domain “Close entourage” in the presence or absence of “Relationship difficulties” (Cohen’s d 0.84; 95% CI 0.51–1.17). Similar observations were made on the 2016 sample (see Additional File 1: Table S4). Cohen’s d was 1.63 (95% CI 1.31–1.95) for the difference in the domain “Material resources” in the presence or absence of “Financial difficulties”, and 1.05 (95% CI 0.69–1.42) for the difference in the domain “Close entourage” in the presence or absence of “Relationship difficulties”. Effect sizes of the differences in the seven QoL subscores in the presence or absence of each of the twenty events are provided in Additional File 1: Table S5 (sample 2011) and 6 (sample 2016). In the 2011 sample, each of the 28 QoL items was most strongly associated with its corresponding domain (see Additional File 1: Table S7), except two items (“Access to health care and prevention” and “Being able to exercise one's creativity, share ideas”) that were highly correlated with other domains as well. The magnitude of the correlations was either large (13 items) or medium (15 items). Results in the 2016 sample were essentially similar (see Additional File 1: Table S8).

**Table 3:** Construct validity: QoL domain subscores in the presence or absence of two stressful events (last 12 months, sample 2011)

Domains	Financial difficulties			Relationship difficulties		
	No	Yes	Effect size	No	Yes	Effect size
	Mean (SD)	Mean (SD)	Cohen’s d (95% CI)^a^	Mean (SD)	Mean (SD)	Cohen’s d (95% CI)^a^
Material resources (N = 1108)	88.8 (18.4)	63.8 (21.5)	1.34 (1.02–1.66)	87.6 (19.4)	78.6 (20.4)	0.46 (0.14–0.79)
Close entourage (N = 1014)	86.8 (20.3)	77.2 (27.9)	0.46 (0.10–0.83)	87.1 (20.5)	69.7 (23.0)	0.84 (0.51–1.17)
Social and cultural life (N = 1047)	85.0 (19.6)	75.3 (25.3)	0.49 (0.17–0.80)	84.9 (19.8)	75.2 (24.5)	0.48 (0.15–0.82)
Esteem and recognition (N = 1089)	83.4 (25.3)	73.9 (31.1)	0.37 (0.08–0.66)	83.3 (25.5)	71.7 (28.2)	0.45 (0.13–0.78)
Health and mobility (N = 1080)	87.0 (21.6)	78.2 (25.0)	0.40 (0.11–0.69)	86.6 (22.0)	83.0 (19.2)	0.17 (− 0.09 to 0.43)
Feeling of safety (N = 1076)	85.4 (20.9)	72.7 (27.1)	0.60 (0.28–0.92)	84.8 (21.4)	81.6 (25.2)	0.15 (− 0.19 to 0.49)
Autonomy (N = 1076)	90.2 (19.9)	80.0 (26.5)	0.50 (0.17–0.82)	90.1 (20.0)	77.7 (27.4)	0.61 (0.22–0.99)

*SD *standard deviation, *CI *confidence interval

^a^Effect size was interpreted as small (> 0.2), medium (> 0.5), or large (> 0.8) [23]

### Weighting procedures

In both 2011 and 2016, medium to large correlations were observed between the single QoL item and both the unweighted QoL score and the four weighted QoL scores (Table 4). The correlations between the unweighted and the four weighted QoL scores were all ≥ 0.96. The moderated regression analysis, regressing the single QoL item on the seven OQoL-7 satisfaction subscores (step 1), additionally on the seven OQoL-7 importance subscores (step 2) and the seven interaction terms of satisfaction by importance (step 3), indicated a significant increase in R^2^ when adding interaction terms of domain satisfaction by importance to the model at step 3 (Table 5). However, the increase in R^2^ was below the small magnitude defined cut-off (ΔR^2^ = 0.018 in both 2011 and 2016 samples).

**Table 4:** Correlations between weighted QoL scores, unweighted QoL score and the single QoL item (Spearman’s rho, 95% confidence interval)

	Sample 2011 (N = 981)		Sample 2016 (N = 982)	
	Single QoL item	Unweighted QoL score	Single QoL item	Unweighted QoL score
Unweighted QoL score	0.47 (0.42; 0.52)	–	0.48 (0.43; 0.52)	–
Weighted QoL score 1	0.48 (0.43; 0.53)	0.99 (0.99; 0.99)	0.47 (0.42; 0.52)	0.99 (0.99; 0.99)
Weighted QoL score 2	0.48 (0.43; 0.53)	0.98 (0.97; 0.98)	0.47 (0.42; 0.52)	0.98 (0.97; 0.98)
Weighted QoL score 3	0.48 (0.43; 0.53)	0.97 (0.97; 0.98)	0.47 (0.42; 0.52)	0.97 (0.97; 0.98)
Weighted QoL score 4	0.47 (0.42; 0.52)	0.96 (0.96; 0.97)	0.46 (0.41; 0.51)	0.97 (0.96; 0.97)

*QoL *quality of life

All correlations were significant (*P* < 0.001)

**Table 5:** Moderated regression analyses for importance QoL subscores

	Single QoL item (sample 2011, N = 851)	Single QoL item (sample 2016, N = 865)
Step 1 (R^2^)	0.175***	0.236***
Satisfaction		
Step 2 (ΔR^2^)	0.045***	0.063***
Satisfaction		
Importance		
Step 3 (ΔR^2^)	0.018**	0.018**
Satisfaction		
Importance		
Satisfaction × importance		

*QoL *quality of life

**P < 0.01;***P < 0.001

## Discussion

There is no gold standard in the measurement of QoL, particularly when attempting to take into account multiple domains of QoL rather than limiting its assessment to specific domains such as health-related QoL. The present study aimed to investigate the validity of the OQoL-7, a multidimensional tool that was developed in the context of a cohort study to assess the QoL of community-dwelling older people in Lausanne, Switzerland. Globally, close to large associations with a single QoL item, as well as small to medium associations with health measures, support the concurrent validity of the OQoL-7 scale in this population. Furthermore, despite the multitude of factors that can influence quality of life in all domains, stressful events during the previous 12 months were associated with QoL subscores in the expected domains. These results provide supportive evidence of the construct validity of the OQoL-7, and complement previous studies that indicated adequate content validity, factorial structure, and internal consistency [14, 24].

The proportion of missing values was acceptable for each of the 28 items except one: “Couples' relationships”. This item was ignored by a large proportion of participants living without partner. Since a given QoL subscore was considered missing if more than one of its constituent items were missing, this resulted in a slightly higher proportion of missing values in the domain “Close entourage” compared to the six other domains. Given that this domain is made of five items, it seems reasonable to allow a second missing item for this particular domain if the item “Couples' relationships” is missing.

Whereas negligible floor effects were observed in the QoL score and in the seven QoL subscores, ceiling effects were more pronounced and deserve particular attention. The proportion of participants who reached the maximal QoL score (15.5% in 2011 and 18.2% in 2016) is at the upper limit of the 15–20% range of values proposed to define a ceiling effect [25, 26]. However, at the domain level QoL subscores showed ceiling effects well above these cut-offs. Respondents’ optimism when assessing their health or their QoL is a common phenomenon that results in left-skewed distributions [27–30]. Ceiling effects observed in five SF-12 dimensions were also reported in previous studies that used the SF-36 [31–33]. To address this issue in future analyses, dichotomizing QoL subscores could appear a simple solution but may not be the best approach. Several statistical techniques have been proposed, such as standard two-part models (e.g. zero-inflated Poisson regression) or joint two-part models [e.g. Tobit regression, generalized linear latent and mixed models (GLLAMM)] [28, 34]. Future work will need to further investigate the feasibility and robustness of these options.

Results from the four weighting procedures that were explored confirm observations from previous studies that reported almost perfect correlations between weighted and unweighted QoL scores [15], and extend previous works that assessed importance weighting using moderated regression but lacked statistical power [16]. Although the inclusion of interaction terms between satisfaction and importance led to an increase in explained variance of the single QoL item, the magnitude of this increase did not reach the cut-off for a small effect size. In future studies, it remains up to the researcher to decide whether the pros of weighting satisfaction by importance (e.g. slight increase in the prediction of QoL) outweigh the cons (e.g. slight increase in missing data).

Several limitations should be mentioned. First, the development of the OQoL-7 and the assessment of its validity were performed in a specific population and context. It should be determined whether the QoL domains covered are relevant in other settings and populations. Second, concurrent validity was tested against instruments that are either unidimensional (single QoL item, self-rated health), or that assess only health-related QoL (SF-12 physical and mental component scores). Unlike multi-item indices, single-item measures do not allow random errors to cancel out, thus increasing variability and decreasing reliability [11]. Further research is required to determine how the OQoL-7 correlates with other multidimensional QoL questionnaires. Third, the OQoL-7 was administered by mailed questionnaire whereas the SF-12 was completed during a face-to-face interview. This difference in administration mode may have influenced the correlations between both assessment tools, as previously reported [35]. Finally, several psychometric properties of the OQoL-7 still need to be tested, such as test–retest reliability and sensitivity to change. A main strength of the present study is however the inclusion of two representative samples of community-dwelling older adults assessed at the same age in 2011 and 2016. This feature of the study design made it possible to show high consistency of the results across these two samples. A further strength is the large sample size, which was adequate to detect small interactions between QoL importance and satisfaction. Despite a large set of potential adjustment variables available, controlled analyses were not performed to avoid over-adjustment for factors that are inherent to quality of life. Furthermore, the study did not aim to demonstrate direct associations between variables.

## Conclusions

The OQoL-7 is a valuable tool to assess the multidimensional QoL of community-dwelling older adults. The decision to use either a QoL score reflecting individuals’ ratings of importance, or a QoL score based only on ratings of satisfaction (unweighted QoL score), depends on the researcher’s priority to either optimize the prediction of QoL, or to limit the number of questions and the amount of missing data.

## Supplementary information

**Additional file 1: Supplementary Method 1**. OQoL-7 scale.** Supplementary Method 2**. OQoL-7 scale (French version).** Supplementary Table S1**. QoL and health characteristics of the 2011 and 2016 samples.** Supplementary Table S2**. Quality of the OQoL-7 data.** Supplementary Table S3**. Quality of the 28 items from the OQoL-7.** Supplementary Table S4**. Construct validity: QoL domain subscores in the presence or absence of two stressful events (last 12 months, sample 2016).** Supplementary Table S5**. Construct validity: Effect size (Cohen’s d) of the difference in QoL domain subscores in the presence or absence of twenty stressful events (last 12 months, sample 2011).** Supplementary Table S6**. Construct validity: Effect size (Cohen’s d) of the difference in QoL domain subscores in the presence or absence of twenty stressful events (last 12 months, sample 2016).** Supplementary Table S7**. Construct validity: correlations between QoL items and domains (sample 2011).** Supplementary Table S8**. Construct validity: correlations between QoL items and domains (sample 2016).

## Data Availability

The authors do not have permission to share data.

## Abbreviations

CI: Confidence interval
SD: Standard deviation
Lc65+: Lausanne cohort 65+
QoL: Quality of life
OQoL-7: Older people Quality of Life-7 domains
SF-12: 12-Item Short Form Health Survey (Medical Outcomes Study)
PCS: Physical component score
MCS: Mental component score
GALES: Geriatric adverse events life scale
ISCED: International Standard Classification of Education
