# Supplementary material for: Validity of the older people quality of life-7 domains (OQoL-7) scale
Source: Health Qual Life Outcomes. 2020 Oct 14;18:340. doi: 10.1186/s12955-020-01589-5 (PMC7557060; doi:10.1186/s12955-020-01589-5)
Supplement: Supplementary file 1 — Additional file 1: Supplementary Method 1. OQoL-7 scale. Supplementary Method 2. OQoL-7 scale (French version). Supplementary Table S1. QoL and health characteristics of the 2011 and 2016 samples. Supplementary Table S2. Quality of the OQoL-7 data. Supplementary Table S3. Quality of the 28 items from the OQoL-7. Supplementary Table S4. Construct validity: QoL domain subscores in the presence or absence of two stressful events (last 12 months, sample 2016). Supplementary Table S5. Construct validity: Effect size (Cohen’s d) of the difference in QoL domain subscores in the presence or absence of twenty stressful events (last 12 months, sample 2011). Supplementary Table S6. Construct validity: Effect size (Cohen’s d) of the difference in QoL domain subscores in the presence or absence of twenty stressful events (last 12 months, sample 2016). Supplementary Table S7. Construct validity: correlations between QoL items and domains (sample 2011). Supplementary Table S8. Construct validity: correlations between QoL items and domains (sample 2016). [file 12955_2020_1589_MOESM1_ESM.pdf]

Supplementary Method 1. OQoL-7 scale

YOUR OPINION ON QUALITY OF LIFE

There are many factors that determine quality of life. For instance, health but also social network or financial resources can influence it. All factors are not equally important for all of us; for instance, living alone can be problematic for some people but not others. In the following questions, we wish to know what is important for your quality of life, and to what extent some factors are problematic.

A. How important are the following factors to your quality of life?

Answer each line

		Very high	Quite high	Quite low	Very low
1.	Financial resources	☐1	☐2	☐3	☐4
2.	Housing comfort	☐1	☐2	☐3	☐4
3.	Sufficient, good quality food	☐1	☐2	☐3	☐4
4.	Friendly atmosphere meals	☐1	☐2	☐3	☐4
5.	Couples' relationships	☐1	☐2	☐3	☐4
6.	Family relationships	☐1	☐2	☐3	☐4
7.	Friendship relationships	☐1	☐2	☐3	☐4
8.	Intergenerational relationships	☐1	☐2	☐3	☐4
9.	Integration into a group, association or society	☐1	☐2	☐3	☐4
10.	Self-esteem	☐1	☐2	☐3	☐4
11.	Being heard and respected	☐1	☐2	☐3	☐4
12.	Cultural and leisure activities	☐1	☐2	☐3	☐4
13.	Not being dependent on help in daily life	☐1	☐2	☐3	☐4
14.	Mobility, being able to travel alone	☐1	☐2	☐3	☐4
15.	Being able to use public transport alone	☐1	☐2	☐3	☐4
16.	Being able to travel	☐1	☐2	☐3	☐4
17.	Physical and mental health	☐1	☐2	☐3	☐4
18.	Access to health care and prevention	☐1	☐2	☐3	☐4
19.	Adequate health insurance coverage	☐1	☐2	☐3	☐4
20.	Safety at home	☐1	☐2	☐3	☐4
21.	Safety in the street	☐1	☐2	☐3	☐4
22.	Religion, philosophy or spiritual life	☐1	☐2	☐3	☐4
23.	Being able to exercise one's creativity, share ideas	☐1	☐2	☐3	☐4
24.	Being able to decide on issues of daily life	☐1	☐2	☐3	☐4
25.	Being able to manage money matters alone	☐1	☐2	☐3	☐4
26.	Being useful to others	☐1	☐2	☐3	☐4
27.	Being able to express one's opinion, to vote, etc.	☐1	☐2	☐3	☐4
28.	Being well informed to meet one's needs and decide	☐1	☐2	☐3	☐4

B. Do you currently feel discomfort or dissatisfaction with any of the following?

Answer each line

		A lot	A little	Not at all
1.	Financial resources	☐1	☐2	☐3
2.	Housing comfort	☐1	☐2	☐3
3.	Sufficient, good quality food	☐1	☐2	☐3
4.	Friendly atmosphere meals	☐1	☐2	☐3
5.	Couples' relationships	☐1	☐2	☐3
6.	Family relationships	☐1	☐2	☐3
7.	Friendship relationships	☐1	☐2	☐3
8.	Intergenerational relationships	☐1	☐2	☐3
9.	Integration into a group, association or society	☐1	☐2	☐3
10.	Self-esteem	☐1	☐2	☐3
11.	Being heard and respected	☐1	☐2	☐3
12.	Cultural and leisure activities	☐1	☐2	☐3
13.	Not being dependent on help in daily life	☐1	☐2	☐3
14.	Mobility, being able to travel alone	☐1	☐2	☐3
15.	Being able to use public transport alone	☐1	☐2	☐3
16.	Being able to travel	☐1	☐2	☐3
17.	Physical and mental health	☐1	☐2	☐3
18.	Access to health care and prevention	☐1	☐2	☐3
19.	Adequate health insurance coverage	☐1	☐2	☐3
20.	Safety at home	☐1	☐2	☐3
21.	Safety in the street	☐1	☐2	☐3
22.	Religion, philosophy or spiritual life	☐1	☐2	☐3
23.	Being able to exercise one's creativity, share ideas	☐1	☐2	☐3
24.	Being able to decide on issues of daily life	☐1	☐2	☐3
25.	Being able to manage money matters alone	☐1	☐2	☐3
26.	Being useful to others	☐1	☐2	☐3
27.	Being able to express one's opinion, to vote, etc.	☐1	☐2	☐3
28.	Being well informed to meet one's needs and decide	☐1	☐2	☐3

Supplementary Method 2. OQoL-7 scale (French version)

VOTRE AVIS SUR LA QUALITÉ DE VIE

Les facteurs qui déterminent la qualité de vie sont nombreux. Par exemple la santé, mais aussi les liens sociaux ou les moyens financiers peuvent l'influencer. Tout n'a pas la même importance pour chacun de nous ; par exemple, être seul·e peut poser problème à certaines personnes et non à d'autres. Dans les questions qui suivent, nous souhaitons connaître ce qui est important pour votre qualité de vie, et dans quelle mesure certains facteurs posent problème.

A. Quelle importance accordez-vous aux facteurs suivants, pour votre qualité de vie ?

Répondre à chaque ligne

		Très grande	Plutôt grande	Plutôt faible	Très faible
1.	Les ressources financières	☐1	☐2	☐3	☐4
2.	Le confort du logement	☐1	☐2	☐3	☐4
3.	Une alimentation de qualité et en suffisance	☐1	☐2	☐3	☐4
4.	L'ambiance conviviale des repas	☐1	☐2	☐3	☐4
5.	Les relations de couple	☐1	☐2	☐3	☐4
6.	Les relations familiales	☐1	☐2	☐3	☐4
7.	Les relations amicales	☐1	☐2	☐3	☐4
8.	Les relations entre générations	☐1	☐2	☐3	☐4
9.	L'intégration à un groupe, une association, une société	☐1	☐2	☐3	☐4
10.	L'estime de soi	☐1	☐2	☐3	☐4
11.	Être écouté·e et respecté·e	☐1	☐2	☐3	☐4
12.	Les activités culturelles et de loisirs	☐1	☐2	☐3	☐4
13.	Ne pas dépendre d'aide dans sa vie quotidienne	☐1	☐2	☐3	☐4
14.	La mobilité, pouvoir se déplacer seul·e	☐1	☐2	☐3	☐4
15.	Pouvoir utiliser les transports publics seul·e	☐1	☐2	☐3	☐4
16.	Pouvoir voyager	☐1	☐2	☐3	☐4
17.	La santé physique et psychique	☐1	☐2	☐3	☐4
18.	L'accès aux soins et à la prévention	☐1	☐2	☐3	☐4
19.	Avoir une bonne assurance pour les frais de santé	☐1	☐2	☐3	☐4
20.	La sécurité chez soi	☐1	☐2	☐3	☐4
21.	La sécurité dans la rue	☐1	☐2	☐3	☐4
22.	La religion, la philosophie, la vie spirituelle	☐1	☐2	☐3	☐4
23.	Pouvoir exercer sa créativité, partager des idées	☐1	☐2	☐3	☐4
24.	Pouvoir décider de sa vie quotidienne	☐1	☐2	☐3	☐4
25.	Pouvoir gérer seul·e ses affaires d'argent	☐1	☐2	☐3	☐4
26.	Être utile aux autres	☐1	☐2	☐3	☐4
27.	Pouvoir exprimer son opinion, voter, etc.	☐1	☐2	☐3	☐4
28.	Être bien informé·e pour faire face à ses besoins et décider	☐1	☐2	☐3	☐4

B. Ressentez-vous actuellement une gêne ou une insatisfaction sur les plans suivants ?

Répondre à chaque ligne

		Beaucoup	Un peu	Pas du tout
1.	Les ressources financières	☐1	☐2	☐3
2.	Le confort du logement	☐1	☐2	☐3
3.	Une alimentation de qualité et en suffisance	☐1	☐2	☐3
4.	L'ambiance conviviale des repas	☐1	☐2	☐3
5.	Les relations de couple	☐1	☐2	☐3
6.	Les relations familiales	☐1	☐2	☐3
7.	Les relations amicales	☐1	☐2	☐3
8.	Les relations entre générations	☐1	☐2	☐3
9.	L'intégration à un groupe, une association, une société	☐1	☐2	☐3
10.	L'estime de soi	☐1	☐2	☐3
11.	Être écouté·e et respecté·e	☐1	☐2	☐3
12.	Les activités culturelles et de loisirs	☐1	☐2	☐3
13.	Ne pas dépendre d'aide dans sa vie quotidienne	☐1	☐2	☐3
14.	La mobilité, pouvoir se déplacer seul·e	☐1	☐2	☐3
15.	Pouvoir utiliser les transports publics seul·e	☐1	☐2	☐3
16.	Pouvoir voyager	☐1	☐2	☐3
17.	La santé physique et psychique	☐1	☐2	☐3
18.	L'accès aux soins et à la prévention	☐1	☐2	☐3
19.	Avoir une bonne assurance pour les frais de santé	☐1	☐2	☐3
20.	La sécurité chez soi	☐1	☐2	☐3
21.	La sécurité dans la rue	☐1	☐2	☐3
22.	La religion, la philosophie, la vie spirituelle	☐1	☐2	☐3
23.	Pouvoir exercer sa créativité, partager des idées	☐1	☐2	☐3
24.	Pouvoir décider de sa vie quotidienne	☐1	☐2	☐3
25.	Pouvoir gérer seul·e ses affaires d'argent	☐1	☐2	☐3
26.	Être utile aux autres	☐1	☐2	☐3
27.	Pouvoir exprimer son opinion, voter, etc.	☐1	☐2	☐3
28.	Être bien informé·e pour faire face à ses besoins et décider	☐1	☐2	☐3

Supplementary Table 1. QoL and health characteristics of the 2011 and 2016 samples

Characteristics	Sample 2011 (N=976)	Sample 2016 (N=963)
**QoL score (OQoL-7)**, mean (SD)	86.7 (16.3)	87.6 (15.7)
**QoL subscores (OQoL-7)**, mean (SD)		
Material resources	87.6 (18.8)	88.3 (18.4)
Close entourage	86.8 (20.3)	86.6 (19.4)
Social and cultural life	84.8 (19.5)	85.7 (19.4)
Esteem and recognition	83.3 (25.0)	84.3 (24.4)
Health and mobility	87.2 (21.3)	87.5 (21.8)
Feeling of safety	85.1 (20.9)	87.3 (19.9)
Autonomy	90.5 (18.9)	91.6 (17.8)
**Single QoL item**, N (%)		
Excellent	79 (8.8)	90 (10.1)
Very good	287 (32.1)	323 (36.1)
Good	480 (53.7)	427 (47.8)
Fair	43 (4.8)	49 (5.5)
Poor	5 (0.6)	5 (0.6)
**Self-rated health**, N (%)		
Very good	97 (9.9)	99 (10.3)
Good	554 (56.8)	549 (57.0)
Average	300 (30.7)	283 (29.4)
Poor	23 (2.4)	30 (3.1)
Very poor	2 (0.2)	2 (0.2)
**SF-12 (PCS)**, mean (SD)	47.9 (8.9)	49.1 (9.1)
**SF-12 (MCS)**, mean (SD)	50.5 (9.0)	49.8 (9.6)

QoL=quality of life

Supplementary Table 2. Quality of the OQoL-7 data

	Sample 2011 (N=1117)			Sample 2016 (N=1091)		
	**Missing**	**Floor**	**Ceiling**	**Missing**	**Floor**	**Ceiling**
**QoL score, %**	1.8	0.1	15.5	1.5	0.0	18.2
**QoL subscores, %**						
Material resources	0.5	0.5	57.2	0.5	0.5	59.6
Close entourage	9.0	1.1	47.7	7.4	0.6	48.9
Social and cultural life	6.0	0.4	45.2	4.0	0.3	48.7
Esteem and recognition	2.2	2.8	60.9	1.8	2.1	62.4
Health and mobility	3.0	1.2	51.1	2.3	1.7	52.4
Feeling of safety	3.3	1.2	45.2	2.4	1.3	51.2
Autonomy	3.3	1.8	61.2	2.2	0.7	66.3

Supplementary Table 3. Quality of the 28 items from the OQoL-7

Items	Sample 2011 (N=1117)			Sample 2016 (N=1091)		
	Missing	Floor	Ceiling	Missing	Floor	Ceiling
01. Financial resources	0.8	4.6	63.4	0.7	5.8	64.3
02. Housing comfort	0.5	3.7	81.9	0.8	2.7	84.1
03. Sufficient, good quality food	1.3	3.3	87.7	0.6	2.6	88.7
04. Friendly atmosphere meals	7.0	4.3	73.8	4.9	4.8	74.5
05. Couples' relationships	25.0	5.1	58.2	23.0	5.2	61.1
06. Family relationships	3.9	4.7	72.1	3.1	3.6	70.6
07. Friendship relationships	3.1	3.7	78.2	2.6	2.6	78.4
08. Intergenerational relationships	4.5	3.5	69.5	3.7	2.7	70.4
09. Integration into a group, association or society	6.5	6.1	65.7	5.8	5.8	66.2
10. Self-esteem	3.9	3.8	68.9	3.0	4.5	68.9
11. Being heard and respected	3.4	5.0	67.5	2.9	3.1	71.2
12. Cultural and leisure activities	5.6	3.2	67.0	4.0	3.9	68.5
13. Not being dependent on help in daily life	3.5	4.3	81.9	3.2	3.4	83.1
14. Mobility, being able to travel alone	2.2	5.0	83.3	2.4	6.0	83.9
15. Being able to use public transport alone	1.8	5.2	84.3	1.9	5.4	86.9
16. Being able to travel	3.6	7.3	71.4	2.8	6.7	73.2
17. Physical and mental health	2.8	5.8	60.6	2.3	6.0	61.2
18. Access to health care and prevention	4.8	2.7	79.7	3.5	2.7	82.5
19. Adequate health insurance coverage	2.9	6.1	75.5	2.4	4.7	77.6
20. Safety at home	3.2	4.8	77.5	2.0	4.3	80.9
21. Safety in the street	3.5	6.7	53.7	2.5	4.9	60.1
22. Religion, philosophy or spiritual life	5.8	3.7	72.4	4.4	3.9	74.8
23. Being able to exercise one's creativity, share ideas	5.1	3.0	70.6	3.9	2.5	74.3
24. Being able to decide on issues of daily life	3.6	4.1	82.5	1.9	3.4	86.3
25. Being able to manage money matters alone	2.6	4.4	85.6	2.5	3.7	86.0
26. Being useful to others	3.7	4.0	73.1	2.6	2.8	77.4
27. Being able to express one's opinion, to vote, etc.	3.0	3.3	82.1	2.2	2.8	85.5
28. Being well informed to meet one's needs and decide	2.8	3.7	79.8	2.3	2.8	82.4

Supplementary Table 4. Construct validity: QoL domain subscores in the presence or absence of two stressful events (last 12 months, sample 2016)

Domains	Financial difficulties			Relationship difficulties		
	No	Yes	Effect size	No	Yes	Effect size
	Mean (SD)	*Mean (SD)*	*Cohen's d (95% CI)* [a]	*Mean (SD)*	*Mean (SD)*	*Cohen's d (95% CI)* [a]
**Material resources (N=1083)**	90.1 (17.0)	61.4 (23.8)	1.63 (1.31 to 1.95)	88.3 (18.7)	79.8 (25.6)	0.44 (0.03; 0.86)
**Close entourage (N=1007)**	87.1 (19.1)	76.8 (24.9)	0.53 (0.22 to 0.83)	87.2 (19.2)	66.8 (21.9)	1.05 (0.69; 1.42)
**Social and cultural life (N=1044)**	85.4 (19.9)	81.3 (21.2)	0.21 (-0.05 to 0.46)	85.5 (19.5)	74.9 (28.7)	0.53 (0.11; 0.96)
**Esteem and recognition (N=1068)**	84.4 (24.4)	76.3 (28.4)	0.33 (0.07 to 0.59)	84.5 (24.5)	69.2 (26.6)	0.62 (0.25; 0.99)
**Health and mobility (N=1063)**	87.7 (21.5)	77.1 (29.8)	0.48 (0.20 to 0.77)	87.3 (22.0)	78.6 (26.6)	0.39 (0.01; 0.78)
**Feeling of safety (N=1062)**	87.6 (19.8)	74.0 (29.0)	0.66 (0.36 to 0.96)	87.0 (20.5)	76.5 (27.6)	0.51 (0.09; 0.93)
**Autonomy (N=1064)**	91.9 (17.6)	82.2 (28.0)	0.53 (0.21 to 0.84)	91.6 (18.3)	82.4 (24.6)	0.50 (0.08; 0.91)

SD=standard deviation; CI=confidence interval

[a] Effect size was interpreted as small (>0.2), medium (>0.5), or large (>0.8) [24]

Supplementary Table 5. Construct validity: Effect size (Cohen's d) of the difference in QoL domain subscores in the presence or absence of twenty stressful events (last 12 months, sample 2011)

Events	Material resources (N=1108)	Close entourage (N=1014)	Social and cultural life (N=1047)	Esteem and recognition (N=1089)	Health and mobility (N=1080)	Feeling of safety (N=1076)	Autonomy (N=1076)
1. Financial difficulties	1.34 ***	0.46 *	0.49 **	0.37 *	0.40 **	0.60 ***	0.50 **
2. New major physical illness	0.12	0.03	0.13	0.09	0.19 *	0.04	0.10
3. Other major physical illness	0.20 *	0.35 **	0.17 *	0.19	0.51 ***	0.19 *	0.30 **
4. Physical illness of close family member	0.12	0.15 *	0.16 *	0.10	0.10	0.04	0.07
5. Accident/injury	0.05	0.01	0.11	-0.01	0.16	-0.01	0.01
6. Marital separation/divorce	0.05	0.30	0.13	0.31	-0.14	0.43	0.52
7. Other marital difficulties	0.60 **	0.98 ***	0.59 **	0.51 **	0.26	0.14	0.68 **
8. Major family problems/conflicts other than with spouse	0.40 **	0.60 ***	0.29 *	0.42 **	0.15	0.36 *	0.13
9. Major problems/conflicts with friends/neighbours	0.46 *	0.69 **	0.34	0.57 **	0.29	0.54 **	0.36
10. Death of spouse	-0.02	0.32	0.09	0.20	0.30	0.07	0.27

Supplementary Table 5. (Continued)

**Events**	**Material resources (N=1108)**	**Close entourage (N=1014)**	**Social and cultural life (N=1047)**	**Esteem and recognition (N=1089)**	**Health and mobility (N=1080)**	**Feeling of safety (N=1076)**	**Autonomy (N=1076)**
11. Death of child	1.49	2.47 *	0.78	1.28	1.67	1.61	1.94
12. Death of parent	-0.35	-0.16	0.66	0.58	0.17	0.13	0.02
13. Death of brother/sister	0.25	-0.01	0.28	0.22	0.29	0.31	0.16
14. Death of other relative/close friend	0.20 *	0.18 *	0.12	0.09	0.05	0.10	0.04
15. Death of pet	0.27	0.06	0.16	-0.04	-0.05	0.19	-0.13
16. Forced to leave/lose home	0.73 *	0.41	0.45	0.36	0.38	0.58	0.53
17. Voluntarily changed place of residence	0.42	0.63	1.06 **	0.43	0.19	-0.06	0.33
18. Difficulty getting adequate professional services	1.42 ***	0.93 *	0.58	0.69	0.73	1.06 ***	1.33 *
19. Victim of crime	0.26	0.26	0.34 *	0.24	0.08	0.19	0.24
20. Became caretaker for relative/friend	0.29 *	0.27 *	0.23	0.26 *	0.01	0.12	0.12

[a] Effect size was interpreted as small (>0.2), medium (>0.5), or large (>0.8) [24]

* P<0.05 ; ** P<0.01 ; *** P<0.001

Supplementary Table 6. Construct validity: Effect size (Cohen's d) of the difference in QoL domain subscores in the presence or absence of twenty stressful events (last 12 months, sample 2016)

**Events**	**Material resources (N=1083)**	**Close entourage (N=1007)**	**Social and cultural life (N=1044)**	**Esteem and recognition (N=1068)**	**Health and mobility (N=1063)**	**Feeling of safety (N=1062)**	**Autonomy (N=1064)**
1. Financial difficulties	1.63 ***	0.53 **	0.21	0.33 *	0.48 **	0.66 ***	0.53 **
2. New major physical illness	0.14	0.10	0.10	0.10	0.25 **	0.05	0.00
3. Other major physical illness	0.36 **	0.10	0.38 **	0.19	0.75 ***	0.31 **	0.33 **
4. Physical illness of close family member	0.03	-0.02	0.14	0.01	-0.07	0.05	-0.02
5. Accident/injury	0.07	0.00	-0.11	-0.14	0.02	0.00	-0.12
6. Marital separation/divorce	0.60	0.88	0.34	0.50	0.49	0.52	0.43
7. Other marital difficulties	0.51	1.18 ***	0.77 **	0.77 ***	0.50 *	0.56 *	0.70 **
8. Major family problems/conflicts other than with spouse	-0.01	0.40 **	0.06	0.30 *	0.11	0.10	0.18
9. Major problems/conflicts with friends/neighbours	1.16 **	0.76 *	0.86 **	0.96 ***	0.86 *	1.28 ***	1.08 **
10. Death of spouse	0.18	0.50	0.03	0.41	0.10	0.10	0.19

Supplementary Table 6. (Continued)

Events	Material resources (N=1083)	Close entourage (N=1007)	Social and cultural life (N=1044)	Esteem and recognition (N=1068)	Health and mobility (N=1063)	Feeling of safety (N=1062)	Autonomy (N=1064)
11. Death of child	1.25 **	0.63	0.42	0.80	1.15	0.86	0.68
12. Death of parent	0.74	0.43	0.95	0.77	0.90	0.76	1.40 *
13. Death of brother/sister	0.17	0.13	0.10	0.20	0.13	0.17	0.27
14. Death of other relative/close friend	0.05	0.10	0.07	0.09	0.11	0.11	0.05
15. Death of pet	0.28	0.05	0.04	0.16	0.49 *	0.15	0.27
16. Forced to leave/lose home	0.11	0.35	0.77	-0.03	0.44	0.33	0.64
17. Voluntarily changed place of residence	0.10	0.08	0.10	0.14	0.16	0.15	0.26
18. Difficulty getting adequate professional services	1.14 **	0.65 *	0.83 ***	0.56	1.30 ***	0.56 **	0.66 *
19. Victim of crime	0.67 *	0.64 **	-0.04	0.12	0.18	0.55 *	0.24
20. Became caretaker for relative/friend	-0.06	0.01	0.10	0.03	-0.03	-0.07	-0.09

[a] Effect size was interpreted as small (>0.2), medium (>0.5), or large (>0.8) [24]

* P<0.05 ; ** P<0.01 ; *** P<0.001

Supplementary Table 7. Construct validity: correlations between QoL items and domains (sample 2011)

Items (Domains)	Material resources	Close entourage	Social and cultural life	Esteem and recognition	Health and mobility	Feeling of safety	Autonomy
(Material resources)							
01. Financial resources (N=970)	0.37	0.22	0.25	0.24	0.21	0.27	0.27
02. Housing comfort (N=972)	0.41	0.31	0.27	0.33	0.31	0.35	0.31
03. Sufficient, good quality food (N=970)	0.40	0.39	0.25	0.35	0.32	0.34	0.36
(Close entourage)							
04. Friendly atmosphere meals (N=965)	0.29	0.46	0.28	0.37	0.31	0.24	0.31
05. Couples' relationships (N=812)	0.28	0.50	0.28	0.38	0.25	0.20	0.30
06. Family relationships (N=971)	0.29	0.59	0.33	0.46	0.30	0.29	0.37
07. Friendship relationships (N=970)	0.32	0.47	0.41	0.46	0.34	0.36	0.43
08. Intergenerational relationships (N=966)	0.24	0.52	0.35	0.46	0.32	0.34	0.37
(Social and cultural life)							
09. Integration into a group, association or society (N=957)	0.23	0.28	0.44	0.36	0.31	0.27	0.33
12. Cultural and leisure activities (N=963)	0.24	0.34	0.49	0.38	0.39	0.33	0.38
22. Religion, philosophy or spiritual life (N=962)	0.16	0.25	0.43	0.29	0.26	0.29	0.33
23. Being able to exercise one's creativity, share ideas (N=966)	0.28	0.43	0.49	0.49	0.41	0.42	0.57

Supplementary Table 7. (Continued)

Items	Material resources	Close entourage	Social and cultural life	Esteem and recognition	Health and mobility	Feeling of safety	Autonomy
(Esteem and recognition)							
10. Self-esteem (N=958)	0.31	0.44	0.43	0.60	0.40	0.37	0.46
11. Being heard and respected (N=958)	0.31	0.55	0.49	0.60	0.38	0.38	0.46
(Health and mobility)							
13. Not being dependent on help in daily life (N=964)	0.28	0.29	0.34	0.35	0.47	0.34	0.43
14. Mobility, being able to travel alone (N=970)	0.24	0.26	0.32	0.31	0.57	0.39	0.43
15. Being able to use public transport alone (N=970)	0.25	0.23	0.34	0.28	0.52	0.39	0.42
16. Being able to travel (N=965)	0.23	0.28	0.40	0.31	0.54	0.46	0.48
17. Physical and mental health (N=970)	0.26	0.33	0.39	0.41	0.52	0.47	0.47
(Feeling of safety)							
18. Access to health care and prevention (N=961)	0.38	0.32	0.39	0.37	0.47	0.44	0.45
19. Adequate health insurance coverage (N=971)	0.42	0.32	0.35	0.34	0.39	0.46	0.43
20. Safety at home (N=965)	0.32	0.35	0.32	0.37	0.40	0.55	0.41
21. Safety in the street (N=965)	0.22	0.29	0.35	0.31	0.42	0.48	0.40

Supplementary Table 7. (Continued)

Items	Material resources	Close entourage	Social and cultural life	Esteem and recognition	Health and mobility	Feeling of safety	Autonomy
(Autonomy)							
24. Being able to decide on issues of daily life (N=967)	0.31	0.36	0.42	0.43	0.47	0.38	0.53
25. Being able to manage money matters alone (N=971)	0.27	0.29	0.30	0.36	0.42	0.39	0.48
26. Being useful to others (N=967)	0.29	0.42	0.43	0.42	0.45	0.39	0.50
27. Being able to express one's opinion, to vote, etc. (N=971)	0.28	0.36	0.40	0.42	0.43	0.42	0.57
28. Being well informed to meet one's needs and decide (N=971)	0.36	0.38	0.45	0.44	0.46	0.49	0.58

Note: for each item, its corresponding QoL subscore was recalculated without the item itself.

Supplementary Table 8. Construct validity: correlations between QoL items and domains (sample 2016)

**Items (Domains)**	**Material resources**	**Close entourage**	**Social and cultural life**	**Esteem and recognition**	**Health and mobility**	**Feeling of safety**	**Autonomy**
(Material resources)							
01. Financial resources (N=975)	0.42	0.31	0.24	0.31	0.30	0.38	0.28
02. Housing comfort (N=978)	0.41	0.34	0.19	0.30	0.31	0.38	0.30
03. Sufficient, good quality food (N=978)	0.42	0.39	0.29	0.36	0.34	0.36	0.39
(Close entourage)							
04. Friendly atmosphere meals (N=977)	0.35	0.52	0.30	0.42	0.32	0.28	0.32
05. Couples' relationships (N=815)	0.28	0.45	0.27	0.36	0.27	0.21	0.26
06. Family relationships (N=976)	0.33	0.58	0.33	0.43	0.30	0.36	0.35
07. Friendship relationships (N=972)	0.32	0.54	0.42	0.44	0.34	0.36	0.43
08. Intergenerational relationships (N=968)	0.27	0.54	0.42	0.41	0.31	0.35	0.37
(Social and cultural life)							
09. Integration into a group, association or society (N=956)	0.16	0.33	0.52	0.40	0.33	0.30	0.36
12. Cultural and leisure activities (N=973)	0.26	0.36	0.54	0.37	0.43	0.35	0.42
22. Religion, philosophy or spiritual life (N=967)	0.13	0.28	0.38	0.27	0.24	0.23	0.35
23. Being able to exercise one's creativity, share ideas (N=969)	0.28	0.42	0.54	0.44	0.40	0.37	0.53

Supplementary Table 8. (Continued)

Items	Material resources	Close entourage	Social and cultural life	Esteem and recognition	Health and mobility	Feeling of safety	Autonomy
(Esteem and recognition)							
10. Self-esteem (N=972)	0.34	0.43	0.45	0.58	0.40	0.38	0.45
11. Being heard and respected (N=972)	0.34	0.51	0.43	0.58	0.41	0.40	0.47
(Health and mobility)							
13. Not being dependent on help in daily life (N=972)	0.31	0.26	0.39	0.33	0.48	0.38	0.45
14. Mobility, being able to travel alone (N=974)	0.30	0.25	0.35	0.34	0.58	0.41	0.43
15. Being able to use public transport alone (N=976)	0.33	0.28	0.36	0.37	0.56	0.43	0.43
16. Being able to travel (N=976)	0.31	0.32	0.44	0.37	0.54	0.41	0.44
17. Physical and mental health (N=975)	0.29	0.37	0.36	0.40	0.51	0.43	0.40
(Feeling of safety)							
18. Access to health care and prevention (N=974)	0.36	0.34	0.36	0.36	0.44	0.42	0.45
19. Adequate health insurance coverage (N=977)	0.45	0.35	0.30	0.37	0.38	0.42	0.40
20. Safety at home (N=977)	0.34	0.30	0.29	0.34	0.39	0.54	0.42
21. Safety in the street (N=973)	0.30	0.32	0.38	0.35	0.40	0.47	0.40

Supplementary Table 8. (Continued)

Items	Material resources	Close entourage	Social and cultural life	Esteem and recognition	Health and mobility	Feeling of safety	Autonomy
(Autonomy)							
24. Being able to decide on issues of daily life (N=976)	0.31	0.36	0.41	0.41	0.44	0.39	0.50
25. Being able to manage money matters alone (N=972)	0.33	0.30	0.36	0.36	0.39	0.43	0.51
26. Being useful to others (N=977)	0.26	0.36	0.47	0.36	0.41	0.35	0.47
27. Being able to express one's opinion, to vote, etc. (N=978)	0.25	0.33	0.44	0.38	0.30	0.36	0.52
28. Being well informed to meet one's needs and decide (N=978)	0.36	0.37	0.38	0.45	0.40	0.46	0.57

Note: for each item, its corresponding QoL subscore was recalculated without the item itself.
